# Ripretinib in combination with tyrosine kinase inhibitor as a late-line treatment option for refractory gastrointestinal stromal tumors: two case reports and literature review

**DOI:** 10.3389/fphar.2023.1122885

**Published:** 2023-05-23

**Authors:** Yefan Zhang, Zhen Huang

**Affiliations:** Department of Hepatobiliary Surgery, National Cancer Center/National Clinical Research Center for Cancer/Cancer Hospital, Chinese Academy of Medical Sciences and Peking Union Medical College, Beijing, China

**Keywords:** case report, gastrointestinal stromal tumor (GIST), refractory GIST, late-line treatment, ripretinib combination therapy

## Abstract

**Background:** This case report presents two clinical cases of metastatic refractory gastrointestinal stromal tumor (GIST) with treatment history of 6–14 years. The follow-up treatment of both cases comprised ripretinib dose escalation and its combination with other tyrosine kinase inhibitors (TKIs). To the best of our knowledge, this is the first report that explored ripretinib combination therapy in the late-line treatment of GISTs.

**Case description: Case-1** represents a 57-year-old female patient who underwent surgical resection for retroperitoneal GIST in 2008. After tumor recurrence in 2009, imatinib was started with complete response for 8 years. Imatinib was followed by sunitinib and regorafenib treatment. In March 2021, due to progressive disease (PD), the patient started ripretinib (150 mg QD) and achieved partial response (PR). Six months later, the patient showed PD. Subsequently, ripretinib dose was increased (150 mg BID) followed by ripretinib (100 mg QD) and imatinib (200 mg QD) combination. CT performed in February 2022 revealed stable lesions with internal visible necrosis. Combination therapy achieved stable disease (SD) for 7 months. On further follow-up in July 2022, the patient showed PD and died in September 2022.

**Case-2:** represents a 73-year-old female patient diagnosed with unresectable duodenal GIST with liver, lung, and lymph node metastases in 2016. After treatment with imatinib, followed by sunitinib, regorafenib, and imatinib rechallenge, ripretinib (150 mg QD) was administered in May 2021, and SD was achieved. Ripretinib dose was increased (200 mg QD) due to PD in December 2021. The tumor showed heterogeneous manifestations, with overall size increase and regression in right posterior lobe. In February 2022, ripretinib (150 mg) plus sunitinib (25 mg) QD was commenced. On follow-up in April 2022, the patient showed slightly improved symptoms with stable hematologic parameters. Combination therapy achieved SD for 5 months and the patient showed PD in July 2022 and discontinued the treatment later. The patient was in poor general condition and was receiving nutritional therapy until last follow-up in October 2022.

**Conclusion:** This case report provides evidence that combination therapy of ripretinib with other TKIs could be an effective late-line treatment option for refractory GIST patients.

## 1 Introduction

Gastrointestinal stromal tumors (GIST) are mesenchymal, heterogeneous groups of tumors originating from interstitial cells of Cajal. Commonly, GISTs emerge in the stomach (∼60% cases) and small intestine (∼25% cases) while rarely occurring in the rectum, colon, esophagus, and other sites (Blay et al., 2021; Qu et al., 2022). KIT proto-oncogene, receptor tyrosine kinase (KIT), or platelet-derived growth factor receptor A (PDGFRA) oncogene mutations are the primary drivers in GIST pathogenesis (Qu et al., 2022). Therefore, treatment with tyrosine kinase inhibitors (TKIs) targeting KIT and PDGFRA, is the standard of care for patients with advanced or metastatic GISTs. Imatinib is the standard first-line treatment for advanced GIST with good clinical efficacy, but a majority of patients eventually progress after a median progression-free survival (mPFS) of 20–24 months (Lostes-Bardaji et al., 2021). TKIs such as sunitinib (in second-line treatment) and regorafenib (in third-line treatment) have been shown to improve the outcomes, however, disease progression still occurs after an mPFS of 5.6 months and 4.8 months, respectively (Demetri et al., 2006; Demetri et al., 2013). Advanced GISTs harbor heterogeneous KIT/PDGFRA mutations, which is a crucial parameter to decide on subsequent treatment (Liegl et al., 2008; Namløs et al., 2018; Bauer et al., 2021).

Ripretinib is a broad-spectrum KIT and PDGFRA switch-control TKI (Smith et al., 2019). The global phase III INVICTUS study has reported a good clinical efficacy for ripretinib as fourth-line (or further line) therapy in patients with advanced GISTs, with mPFS of 6.3 months versus 1.0 months, overall response rate (ORR) of 11.8% versus 0%, and the median overall survival of 18.2 months versus 6.3 months when compared with placebo (Blay et al., 2020; M; von Mehren, 2021). Ripretinib was also associated with a favorable and tolerable safety profile where the most common treatment-related adverse events (AEs) were of grade 1–2 (Blay et al., 2020). Further, a Chinese bridging study of INVICTUS study has reported that the efficacy and safety data of ripretinib in the Chinese population are very consistent with the global population, with a mPFS of 7.2 months and an ORR of 18.4% (Li et al., 2022). Based on these outcomes, ripretinib was approved as the ≥fourth-line treatment of advanced metastatic GISTs in multiple countries around the world including China. Currently, there is no standard therapy available following the progression on fourth-line treatment. Various TKIs such as dasatinib and nilotinib have been investigated as potential treatments in late-line therapy, along with imatinib rechallenge, but with limited clinical benefit (Sawaki et al., 2011; Trent et al., 2011; Kang et al., 2013). Hence, there is an urgent unmet need for innovative strategies to effectively inhibit tyrosine kinase receptors after disease progression on standard therapies.

In this article, we report two clinical cases of refractory GIST treated with ripretinib after progressing on multi-line treatments. Both patients achieved good efficacy (partial response [PR] or stable disease [SD]) with tolerable AEs after the ≥fourth-line treatment with ripretinib. After disease progression, the follow-up treatment of these two patients was carried out by increasing the dose of ripretinib and exploring the combination of ripretinib with other TKIs. Both patients achieved SD for 5–7 months with ripretinib combination therapy. To the best of our knowledge, this is the first time that ripretinib combination therapy has been explored in the late-line treatment of GISTs. The findings are presented and discussed in this article.

## 2 Case presentations

### 2.1 Case 1 presentation

**FIGURE 1:**
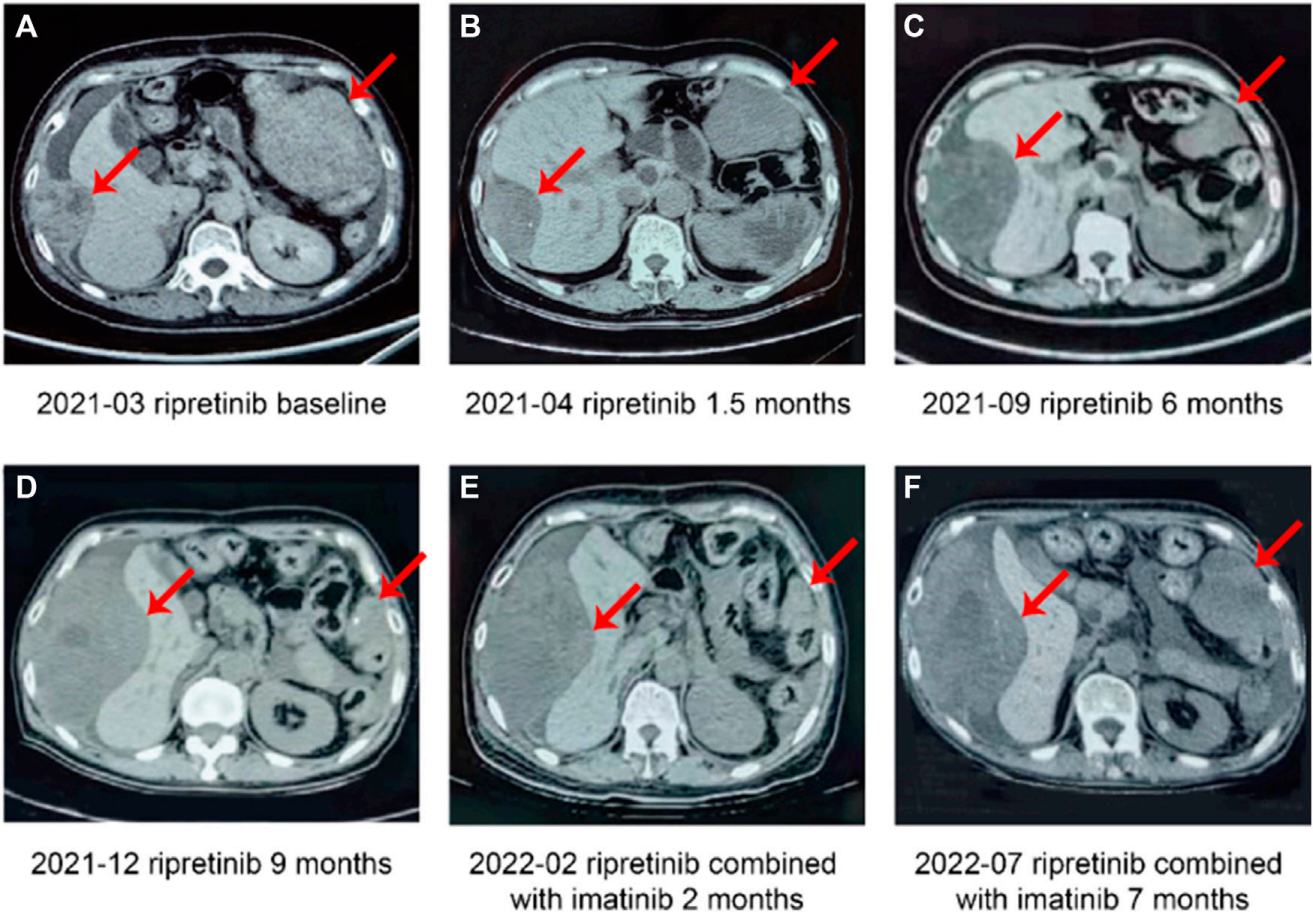
Case 1, a case of multiline treatment (retroperitoneal) for stromal tumor—summary of imaging data of the fourth-line and subsequent treatment. **(A)** Patient newly started on ripretinib (baseline). **(B)** Partial response after 1.5 months of ripretinib treatment from baseline. **(C)** Disease progression after 6 months of ripretinib treatment from baseline. **(D)** Disease progression after 9 months of ripretinib treatment from baseline. **(E)** Stable disease after 2 months of treatment with ripretinib and imatinib combination. **(F)** Disease progression after 7 months of ripretinib and imatinib combination treatment.

In September 2008, a 57-year-old female underwent surgical resection of a 30-cm retroperitoneal stromal tumor at our hospital [Immunohistochemical (IHC): CD117 (+), CD34 (+), Ki67 (Hotspot area 25%+), DOG1 (+), S-100 (−), SMA (−)]. Postoperatively, the patient did not receive any TKIs. In October 2009, a re-examination revealed liver and spleen metastases along with enlarged lymph nodes. Therefore, reoperation (R2 surgery) was performed and the patient was started on imatinib 400 mg once daily QD postoperatively till October 2014. Following this treatment, the patient achieved a complete response as defined by Response Evaluation Criteria in Solid Tumors (RECIST) 1.1 (Eisenhauer et al., 2009) and hence, imatinib was discontinued for 1 year till October 2015. A computed tomography (CT) and magnetic resonance (MR) examination in October 2015 revealed the presence of multiple nodules in the abdominal cavity, the right lobe of the liver and the left adrenal region, suggesting a strong possibility of metastases and multiple liver cysts. Genetic testing detected an imatinib-sensitive KIT exon 11 gene deletion (p.W557_K558del) and hence imatinib 400 mg QD treatment was restarted, and tumor progression was controlled. In October 2018, the patient presented with multiple liver mass, gallbladder stones, and cystic and solid mass in the spleen area which were evaluated as progressive disease (PD). Subsequently, the dose of imatinib was increased to 600 mg/day. Four months later, a follow-up examination in February 2019 revealed progression of abdominal and hepatic subcapsular lesions indicating PD and hence second-line sunitinib 37.5 mg QD was commenced. The patient voluntarily stopped the medication for 7 days within 2 weeks of treatment initiation due to proteinuria. Thereafter, the patient was counseled and recommenced on sunitinib till October 2020 when a CT scan revealed a smaller abdominal tumor but larger liver metastases indicating PD. Considering the PD and side effects (serious proteinuria) associated with sunitinib, the treatment was switched to third-line regorafenib 80 mg QD. Three months later, in January 2021, CT revealed left abdominal tumor and liver metastases were larger than before with a 102 mm × 55 mm mass in the left upper abdominal region and a 44 mm × 38 mm right subphrenic nodule at the outer edge of the liver indicating PD. Hence, regorafenib was discontinued and ripretinib 150 mg QD treatment was started on 13 March 2021 and a PR was achieved 46 days later on 28 April 2021 as evident by significantly reduced internal density of the left abdominal mass and reduced diameter of the lesion between the right liver and the diaphragm on CT indicating obvious cystic degeneration (Supplementary Figure S1; Figures 1A, B). The perihepatic effusion had completely disappeared and the patient’s symptoms also improved significantly. No serious adverse event was observed.

In September 2021, CT showed lesion of left upper quadrant shrank further, but the lesion of the right liver capsule was enlarged which were evaluated as disease progression (Figure 1C). The genetic testing revealed the presence of KIT 11, 13, and 17 exon mutations (KIT 11 W557_K558del, P573R, KIT 13 V654A, KIT 17 C809G, Y823S). Subsequently, ripretinib dose was increased to 150 mg twice a day (BID) that patient was able to tolerate with manageable side effects. Three months later (December 2021), CT showed lesion of left upper quadrant shrank further, but lesion of right liver capsule significantly enlarged indicating PD (Figure 1D) and was started on combination therapy of ripretinib 100 mg QD + imatinib 200 mg QD based on genetic tests, clinical efficacy, and tolerability profile of prior drug treatments along with multidisciplinary team discussion. The patient showed stable lesions with necrosis on a follow-up examination after 2 months in February 2022 indicating a SD (Figure 1E). Patient experienced mild fatigue (grade 1) and alopecia. No abnormalities in laboratory parameters were observed.

**FIGURE 2:**
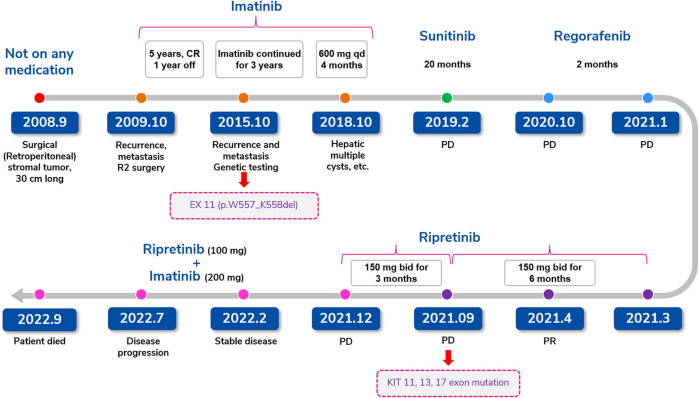
Treatment summary for case 1 (retroperitoneal stromal tumor).

However, CT examinations in July 2022 revealed significantly enlarged lesions indicating PD (Figure 1F) and discontinued the TKI therapy later. Unfortunately, the patient died due to abdominal distension and nutritional problems in September 2022. The complete timeline of the treatment is illustrated in Figure 2.

### 2.2 Case 2 presentation

In June 2016, a 73-year-old female with multiple liver lesions identified on ultrasonography visited our hospital. Liver biopsy revealed stromal tumor [IHC: CD117 (+), CD34 (+), Ki67 (5%+), DOG1 (+), S-100 (−), SMA (−)], which was further confirmed as metastatic unresectable duodenal GIST upon positron emission tomography-CT (PET-CT) examination with liver, lung, and lymph node metastases. The patient was started on imatinib 400 mg QD, which was reduced to 300 mg QD due to AEs (skin pruritus, myelosuppression, and gastrointestinal toxicity) resulting in a SD that was maintained for the next 2 years.

In September 2018, PD was identified based on a CT review; hence, imatinib was discontinued and replaced by sunitinib 37.5 mg QD (2 weeks on treatment followed by 1 week off) as second-line therapy. In June 2019, genetic testing revealed a KIT exon 11 gene deletion (p.W557_K558del) and an exon 13 mutation (pV654A). Six months later, in January 2020, the patient presented with larger liver metastases and a larger tumor between the liver and stomach compared with previous reports. The findings were evaluated as PD and subsequently, the dose of sunitinib was increased to 50 mg QD (4 weeks on treatment followed by 2 weeks off).

In February 2020, genetic testing revealed the presence of KIT exon 11, exon 13, and TP53 mutations. In July 2020, CT revealed liver metastases progression indicating PD. Hence, sunitinib was discontinued and the patient was administered third-line regorafenib 120 mg QD (3 weeks on treatment followed by 1 week off). However, 2 months later in September 2020, the patient still had a PD as revealed by further enlarged tumor size on CT. A month later, in October 2020, regorafenib was discontinued. In November 2020, CT showed a significantly smaller tumor in the portal space and highly heterogeneous lesions in multiple liver metastases and imatinib was re-commenced (re-challenge).

**FIGURE 3:**
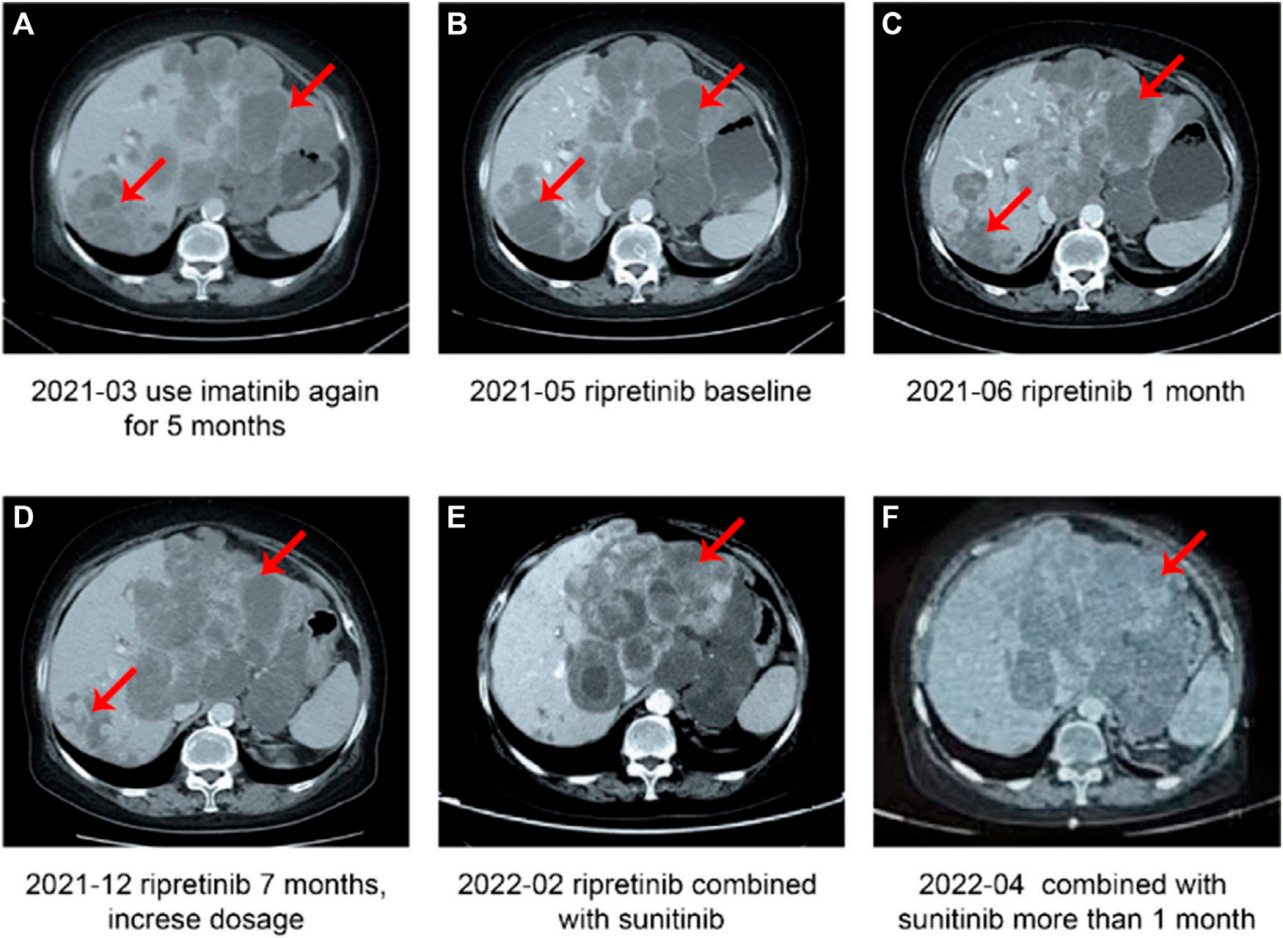
Case 2, a case of duodenal stromal tumor with multiple metastases treated with multiple lines—summary of image data of the fourth-line and subsequent treatment. **(A)** Disease progression after 5 months of treatment with imatinib. **(B)** Patient newly started on ripretinib (baseline). **(C)** Stable disease after 1 month of treatment with ripretinib. **(D)** Disease progression after 7 months of ripretinib treatment from baseline. **(E)** Disease progression after 2 months of increased ripretinib dose. **(F)** Stable disease after treatment with ripretinib and sunitinib combination.

In March 2021, the patient showed presence of the new tumor around the cardia indicating metastasis (Figure 3A) and a PD. Hence, imatinib was discontinued and patient was started on ripretinib 150 mg QD as the fifth-line treatment in May 2021. Before starting on ripretinib, the patient had a large tumor burden and compression around the cardia (Figure 3B). CT after 1 month of treatment with ripretinib (June 2021) showed a reduced tumor size, whereas the multiple liver metastases had partially increased in a few regions and decreased in others indicating reduced tumor load (Figure 3C). The response was evaluated as SD. The symptoms of the patient were relieved, and no serious AEs were observed.

In November 2021, a KIT exon 11 gene deletion (p.W557_K558del) and an exon 13 mutation (pV654A) were detected. In December 2021, a CT examination revealed highly heterogeneous lesions, with right posterior lobe lesion shrinkage and a change in the shape of the tumor around the cardia and multiple enlarged liver metastases indicating PD (Figure 3D). After careful consideration of the patient’s advanced age, ripretinib dosage was increased to 200 mg QD. However, in February 2022, tumors still showed heterogeneous manifestations, with overall size increase and regression in right posterior lobe (Figure 3E). Since there are no recommended therapeutic options available for the late-line treatment of such progressive cases, a combination of ripretinib 150 mg QD + sunitinib 25 mg QD was started after considering patient’s previous genetic testing results and discussion with the hospital’s multidisciplinary team. A follow-up examination in April 2022 showed slightly improved symptoms with stable blood parameters (Figure 3F) suggesting a SD. Patient had mild fatigue (grade 1-2). No abnormalities in laboratory parameters were observed. However, in July 2022, the patient had a disease progression and discontinued the TKI therapy later. The patient was in poor general condition and receiving nutritional therapy until the last follow-up in October 2022.

**FIGURE 4:**
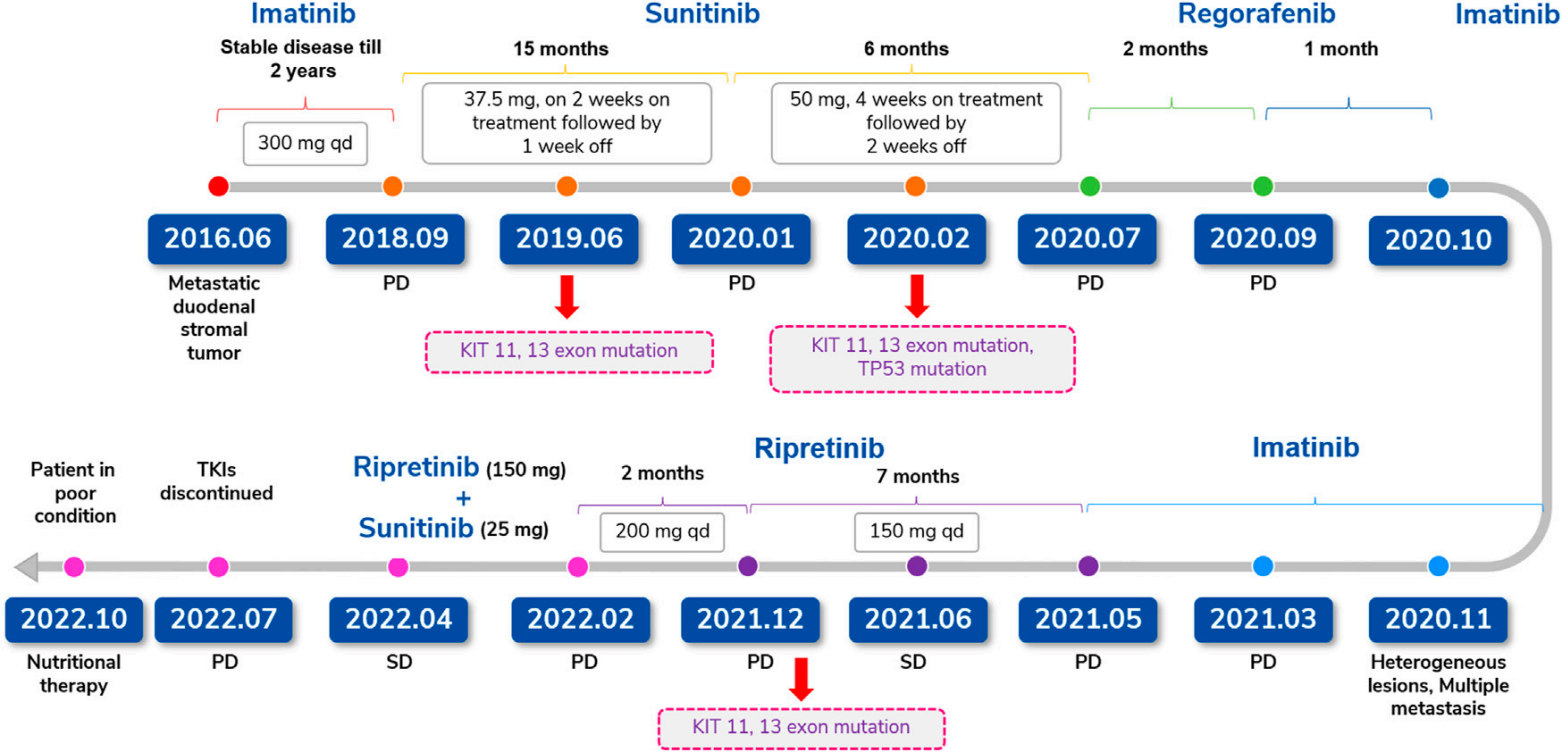
Treatment summary for case 2 (duodenal stromal tumor with multiple metastases).

The complete timeline of the treatment is illustrated in Figure 4.

## 3 Discussion

Currently, there is no standard therapy available for patients with advanced GISTs after fourth-line or above ripretinib treatment. Hence, there is a major unmet clinical need for selecting an appropriate follow-up treatment. Ripretinib 150 mg BID is recommended by the National Comprehensive Cancer Network guidelines as an additional option for patients who have progressed on approved standard therapies. In this study, ripretinib 150 mg BID or dose escalation was used after progression on approved therapies, which resulted in delaying disease progression to a certain extent. Importantly, both case studies provided evidence on the efficacy of ripretinib and sunitinib/imatinib combination as a therapeutic option for arresting GIST progression in selective patients, who are refractory to the current standard therapies. To the best of our knowledge, this is the first time that ripretinib combination therapy has been explored in the late-line treatment of GISTs.

TKIs targeting KIT and PDGFRA have dramatically improved clinical outcomes in patients with metastatic GIST. However, secondary mutations typically arise during or after the progression on first-line imatinib treatment, conferring resistance against the therapeutic agent. These mutations are primarily concentrated in the adenosine triphosphate (ATP)-binding pocket and activation loop region of KIT/PDGFRA, posing a challenge in choosing the subsequent therapies (Liegl et al., 2008; Blay et al., 2021). Sunitinib and regorafenib inhibit some TKI resistance mutations but are relatively ineffective in inhibiting others (Heinrich et al., 2008; Serrano et al., 2019b). Moreover, development of separate resistant clones which harbor different resistance mutations lead to a relatively short disease control in second- and third-line treatments for GIST (Antonescu et al., 2005; Wardelmann et al., 2005; Heinrich et al., 2008; Serrano et al., 2019b). Ripretinib, a broad-spectrum KIT and PDGFRA switch-control TKI (Smith et al., 2019), has shown significant clinical activity and a favourable safety profile as ≥ fourth-line treatment in patients with advanced GISTs. At present, the mechanism of tumor resistance to ripretinib is unknown. Further, in heavily pretreated GISTs, intra- and inter-tumor mutational heterogeneity is exacerbated, resulting in treatment bottlenecks. Therefore, combination therapy, in addition to participating in new drug clinical trials, may bring new hope to the patients with refractory GISTs.

The combination therapy is currently being investigated in clinical research and is possibly a future trend for advanced GIST. TKI combined with KIT/PDGFRA downstream pathway inhibitors, such as imatinib combined with MEK162 (binimetinib), and ripretinib combined with MEK inhibitor (trametinib) demonstrate the potential to improve clinical outcomes in patients with advanced GISTs (Chi et al., 2020; Gupta et al., 2021). Furthermore, because of the difference in TKIs’ sensitivity to GIST secondary mutations and their mechanism of action, different TKI combination therapies are also presently being explored, such as sunitinib alternating with regorafenib (Serrano et al., 2019a), and PLX9486 combined with sunitinib (Wagner et al., 2021). These studies offer suggestions for the application of combination therapy in patients with advanced GIST. However, further research and exploration are warranted to comprehensively investigate the efficacy, drug safety, and patient tolerance in combination therapy.

In contrast to the mechanism of action of the first 3 lines of treatment, ripretinib, a switch-control TKI, is designed to bind with both the switch pocket and the activation switch to lock the kinase in the inactive state. This binding prevents downstream signaling and cell proliferation, consequently providing broad inhibition of KIT and PDGFRA kinases rendered constitutively active by both primary and secondary mutations that lead to drug-resistant GISTs (Smith et al., 2019; Bauer et al., 2021). Ripretinib will be the preferred backbone for future combination therapy strategies, because of its broad range of KIT oncoproteins inhibition, clinical significance, maintenance of physical function and health status, and in historical comparison; a favorable safety and toxicity profile (Serrano and Bauer, 2022).

In this report, both patients achieved good efficacy (PR or SD for 6–7 months) with a favorable safety profile after the ≥fourth-line treatment with ripretinib. During the ripretinib dose escalation, some lesions reduced in size while others enlarged, indicating that ripretinib was still effective against certain lesions. Genetic testing was performed following PD on both patients, and associated KIT mutations were detected. Hence, subsequent combination therapy was considered and ripretinib was used as a component in the combination due to its acceptable safety profile.

The combination of ripretinib and sunitinib showed good efficacy in the treatment of case- 2. Sunitinib, a multitargeted TKI, binds with the ATP-binding domain of both KIT and PDGFRA, and has a strong antiangiogenic activity (Demetri et al., 2006), which may have a synergistic effect with ripretinib. Hence, this combination therapy may become an important way to control multi-resistance in GISTs. However, the safety profile of the drug must also be considered in combination therapy, because case 1 patient experienced severe proteinuria during the treatment with sunitinib. Hence, a new attempt was made in which ripretinib was combined with imatinib for treating the patient in case-1. This decision was made based on the genetic testing, clinical efficacy, and tolerability characteristics of previous drug treatment and after a multidisciplinary team discussion. The disease was controlled for some time. TKIs were used as a combination in first- and third-generation epidermal growth factor receptor-TKI therapy on patients with non-small cell lung cancer (Niederst et al., 2015) which gave us some ideas to use the combination drugs in our 2 patient cases. However, the mechanism of action remains unclear and warrants further investigation. We look forward to exploring the mechanism of resistance against ripretinib which will provide further guidance on subsequent treatment. In our study, the combination regimens had a curative effect, and both patients achieved SD with an acceptable safety profile. Although both patients endured PD within 5–7 months of combination therapy, these findings supplement the evidence for the use of combination therapy in patients progressing on standard therapies. In the future, more research and data support will be required to further substantiate the use of such regimens.

In summary, the approval of new drugs has expanded the armamentarium available for the treatment of advanced GISTs. Furthermore, the lack of effective therapy in patients with advanced GISTs after progression on standard treatment has led to the testing of new combination of drugs, which will help to prolong the survival and improve the quality of life of these patients. Both, case 1 and case 2 evidenced the combination of ripretinib with other TKIs such as sunitinib and imatinib as an effective therapeutic option for arresting the progression of GIST in selective patients who are refractory to current standard therapies. The mechanism of action remains unclear and requires further investigations.

## Data Availability

The original contributions presented in the study are included in the article/Supplementary Material, further inquiries can be directed to the corresponding author.
