# Supplementary material for: Ripretinib in combination with tyrosine kinase inhibitor as a late-line treatment option for refractory gastrointestinal stromal tumors: two case reports and literature review
Source: Front Pharmacol. 2023 May 23;14:1122885. doi: 10.3389/fphar.2023.1122885 (PMC10242384; doi:10.3389/fphar.2023.1122885)
Supplement: Supplementary file 1 [file Image1.PDF]

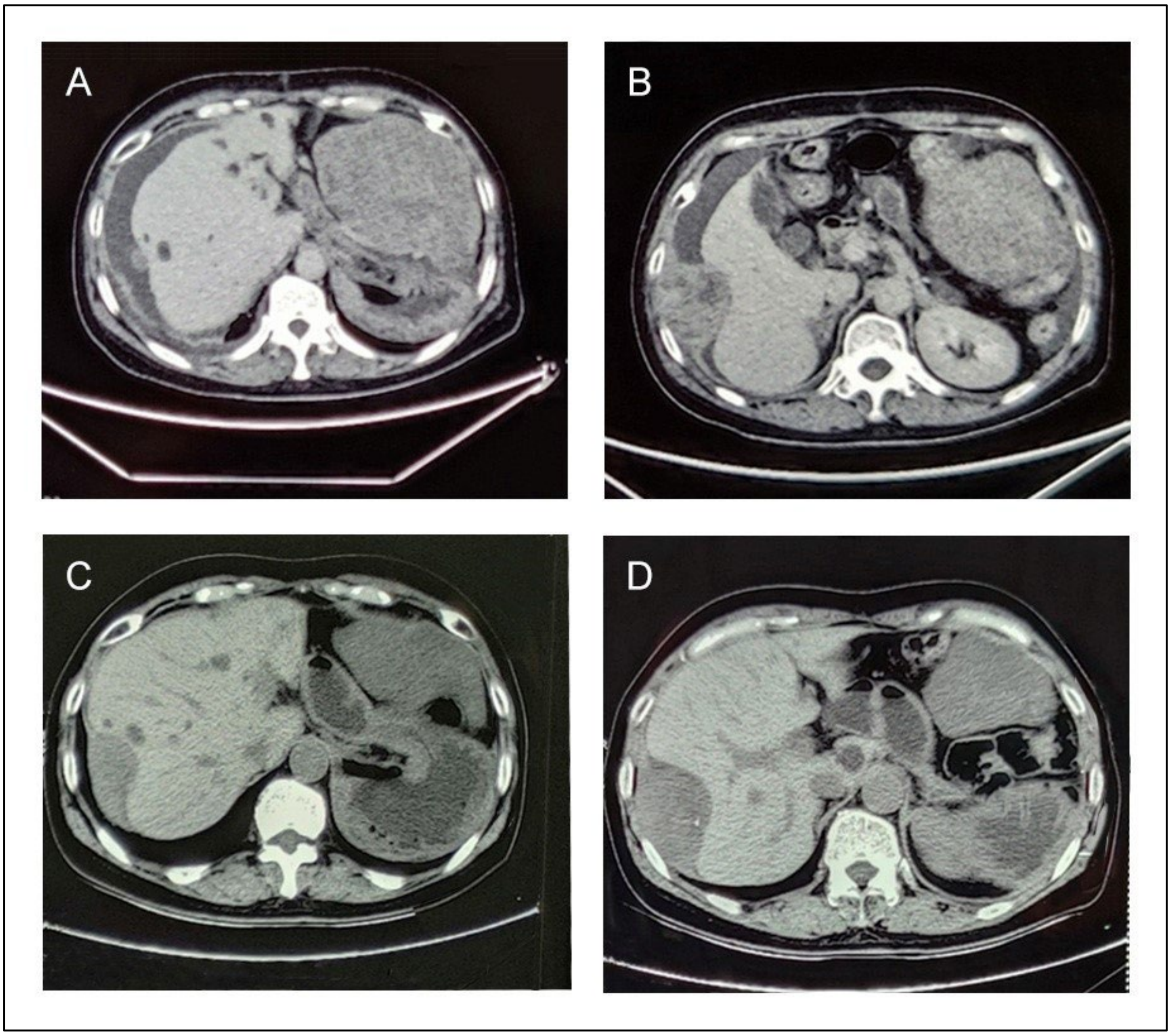

SUPPLEMENTARY FIGURE S1.
(A) and (B): - CT at baseline before starting ripretinib on March 9 2021 (left upper quadrant: 13.7 cm*11.5 cm; right liver capsule: 7.0 cm*6.6 cm).
(C) and (D): - CT at 46 days of ripretinib April 28 2021 (left upper quadrant: 10.2cm*5.2cm; right liver capsule: 5.0cm*4.7cm).
